# Changes in DNA methylation profiles of myalgic encephalomyelitis/chronic fatigue syndrome patients reflect systemic dysfunctions

**DOI:** 10.1186/s13148-020-00960-z

**Published:** 2020-11-04

**Authors:** A. M. Helliwell, E. C. Sweetman, P. A. Stockwell, C. D. Edgar, A. Chatterjee, W. P. Tate

**Affiliations:** 1 Department of Biochemistry, University of Otago, Dunedin, 9016 New Zealand; 2 Department of Pathology, University of Otago, Dunedin, 9016 New Zealand

**Keywords:** ME/CFS, DNA methylation, RRBS, DMAP, MethylKit, Epigenetics

## Abstract

**Background:**

Myalgic Encephalomyelitis/Chronic Fatigue Syndrome (ME/CFS) is a lifelong debilitating disease with a complex pathology not yet clearly defined. Susceptibility to ME/CFS involves genetic predisposition and exposure to environmental factors, suggesting an epigenetic association. Epigenetic studies with other ME/CFS cohorts have used array-based technology to identify differentially methylated individual sites. Changes in RNA quantities and protein abundance have been documented in our previous investigations with the same ME/CFS cohort used for this study.

**Results:**

DNA from a well-characterised New Zealand cohort of 10 ME/CFS patients and 10 age-/sex-matched healthy controls was isolated from peripheral blood mononuclear (PBMC) cells, and used to generate reduced genome-scale DNA methylation maps using reduced representation bisulphite sequencing (RRBS). The sequencing data were analysed utilising the DMAP analysis pipeline to identify differentially methylated fragments, and the MethylKit pipeline was used to quantify methylation differences at individual CpG sites. DMAP identified 76 differentially methylated fragments and Methylkit identified 394 differentially methylated cytosines that included both hyper- and hypo-methylation. Four clusters were identified where differentially methylated DNA fragments overlapped with or were within close proximity to multiple differentially methylated individual cytosines. These clusters identified regulatory regions for 17 protein encoding genes related to metabolic and immune activity. Analysis of differentially methylated gene bodies (exons/introns) identified 122 unique genes. Comparison with other studies on PBMCs from ME/CFS patients and controls with array technology showed 59% of the genes identified in this study were also found in one or more of these studies. Functional pathway enrichment analysis identified 30 associated pathways. These included immune, metabolic and neurological-related functions differentially regulated in ME/CFS patients compared to the matched healthy controls.

**Conclusions:**

Major differences were identified in the DNA methylation patterns of ME/CFS patients that clearly distinguished them from the healthy controls. Over half found in gene bodies with RRBS in this study had been identified in other ME/CFS studies using the same cells but with array technology. Within the enriched functional immune, metabolic and neurological pathways, a number of enriched neurotransmitter and neuropeptide reactome pathways highlighted a disturbed neurological pathophysiology within the patient group.

## Introduction

Myalgic Encephalomyelitis/Chronic Fatigue Syndrome (ME/CFS) is a poorly understood disease estimated to affect an average of 0.89% [1] of the global population, though estimates have been higher such as 7%, as has been observed in Iceland [2]. Core symptoms include severe fatigue, post-exertional malaise, and cognitive, sleep and orthostatic dysfunctions as well as additional debilitating symptoms that collectively are often severe enough to leave patients house or bedbound. There is enormous emotional, social and financial strain on the affected individual and their families [3].

The acute phase of ME/CFS can be sustained long term in ~ 25% of cases, but in the remaining ~ 75% the disease progresses to a lifelong chronic phase that is generally low functioning. A small percentage (~ 5%) may recover although this subgroup may have originally been misdiagnosed [4]. Presentation of ME/CFS symptoms varies among affected individuals and along each person’s disease course, with regular periods of relapse and partial recovery occurring over time. The disease presentation and current research implicates multi-systemic dysfunction including: metabolic, neurological and immune/inflammatory disruptions. Recent work has stressed the role of the mitochondria in particular with a proposed dysfunction resulting in an overall state of decreased energy metabolism unable to compensate for stress events [5]. The disease onset itself often follows a major ‘stress’ event, with a viral infection commonly reported by patients. This has established the hypothesis that onset of ME/CFS requires a stressor event coupled with a normally ‘silent’ component of genetic risk [3].

A number of environmental factors such as exposure to toxins/chemicals, stressful life events and especially viral infections have been frequency reported in association with the onset and progression of ME/CFS [3]. As environmental factors such as these have been observed to cause changes to an individual’s DNA methylation, this has prompted investigations into whether there are specific epigenetic changes linked to the disease [6–10]. DNA methylation is the best understood epigenetic modification where the addition of a methyl group to the cytosine base of DNA is associated with changes in gene expression without altering the genomic code itself [11]. This biological interface between an individual’s environment and the regulation of their genome can provide insights into an individual’s disease activity.

A small number of studies [6–10] have utilised array-based technology to interrogate DNA methylation in ME/CFS patients at a large number of sites across the genome. There are strengths in array-based genome scale investigation since the targeted probes result in low experimental variability, but they do have a restricted CpG coverage as they are designed to target regions that capture RefSeq genes and promoter CpG islands. Reduced representation bisulphite sequencing (RRBS), used in the current investigation, explores more CpG regions across the genome, although the technology can have greater technical variability, for example because of ‘uneven read depths’ and ‘coverage of CpG regions’ among libraries [12, 13]. The array-based investigations into ME/CFS [6–10] have identified a number of changes with some similarities among the studies such as identification of overrepresented functional pathways linked to cell activity and immune functions. However, there are also many variations within the approaches and analyses, resulting in significant differences in outcomes from these published studies.

A specific focus of a number of investigations into the methylation status of ME/CFS has been on the potential association between DNA methylation abnormalities and the activity of the hypothalamic–pituitary–adrenal (HPA) axis. The HPA axis is involved in a number of systems maintaining circadian patterns, homeostatic regulations and in an individual’s response to stress where environmental cues stimulate the HPA system. This results in the release of glucocorticoids such as cortisol producing a corresponding response in a large number of systems including: metabolism, immune, reproductive, nervous and cardiovascular systems. Studies have observed irregular HPA activity in patients when exposed to challenges such as the Trier Social Stress test [14] and dexamethasone [8]. This supports the irregular activity of the HPA axis as a basis for patients’ disproportional immune response to stress. Key work supports an epigenetic element with the identification of a number of differentially methylated sites associated with glucocorticoid sensitivity in patients [8].

By investigating the epigenetic patterns distinct to ME/CFS patients compared to matched controls, the overall multi-systemic disruption caused by the disease can be better understood. This study aimed to identify changes in the PBMC DNA methylation of a New Zealand cohort of ME/CFS patients. This included identification of differentially methylated genes and regulatory regions linked to functional pathways.

## Results

### Generating sequencing-based DNA methylome maps for ME/CFS patients and age-matched healthy controls

**Fig. 1:**
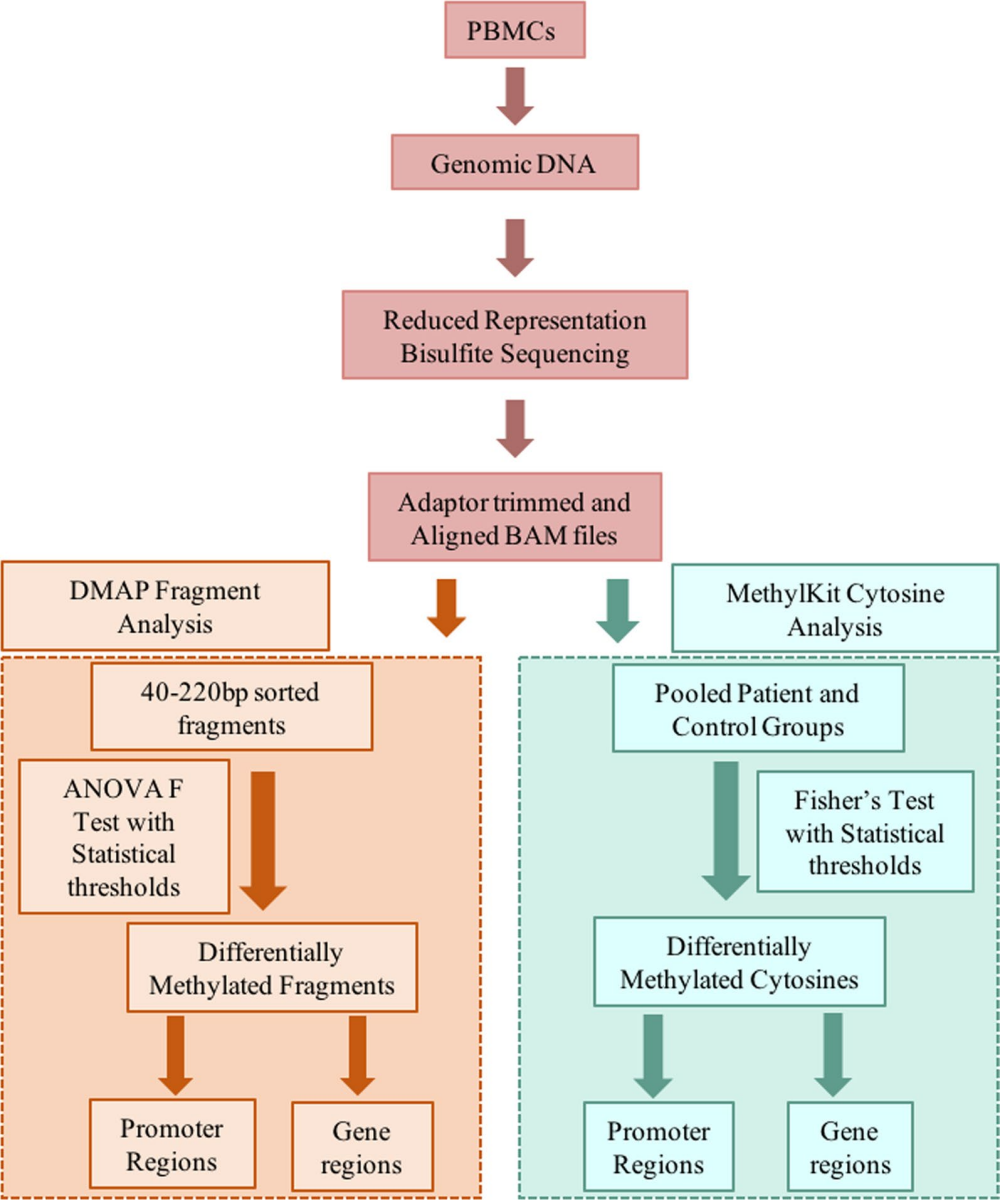
The study and analysis workflow for ME/CFS patients and age-matched healthy control methylome: DNA was isolated from the Peripheral blood mononuclear cells (PBMCs) of 10 ME/CFS patients and 10 age/sex matched healthy controls. DNA was processed to produce reduced representation bisulphite sequencing libraries. This DNA was then sequenced and the data aligned and trimmed using Bismark. The aligned RRBS sequence data were then analyzed using two parallel analyses pipelines: DMAP and MethylKit. DMAP utilised an ANOVA F test comparing patients and controls (requiring at least seven from each group to be present in each comparison between fragments). Associated promoter and gene regions were then identified. Methylkit analysis pooled the patient and control groups before comparison using a Fisher’s test to identify differentially methylated cytosines (requiring at least seven from each group to be present in the comparison between cytosines). Promoter and gene regions were then isolated and investigated

Ten ME/CFS patients and ten age/sex matched controls were investigated to identify patterns of DNA methylation specific to the disease state. PBMCs were purified from each subject and genomic DNA isolated for library preparation for reduced representation bisulphite sequencing (RRBS). Following the sequencing, the data were aligned to the human genome hg19 using Bismark [15] and analyzed following two independent analysis approaches. The first was the utilisation of DMAP [16, 17] with an ANOVA F test comparison that identified changes in sequence data on 40–220 bp DNA fragments in order to capture the DNA methylation patterns across fragment lengths produced in the process of creating the RRBS libraries. The second approach involved the MethylKit pipeline [18] to investigate changes between individual CpGs utilising a Fisher’s test comparing DNA methylation between patients and the controls. This could detect any additional changes not observed in the fragment analysis. Figure 1 describes the study and analysis workflow.

A previous RRBS study has indicated that using either single CpG analysis or fragment-based methylation patterns as an independent method was a valid way to analyze the data [19]. We have analyzed our RRBS methylome here by both approaches to provide additional technical validation to our study.

A summary of the sequencing data and Bismark alignment output is shown in Table 1. Most of the methylation occurs in CpGs with a range of 40–60% across the cohort, and there was only a small percentage of non-CpG methylation. For the patients and controls, the mapping efficiency was variable with about half the cohorts at 40% or above. The bisulphite conversion rate was calculated to be > 99%.

**Table 1:** Summary of sequencing data and Bismark alignment output

Sample ID	Total reads	Map. effic %	MethylC CpG %	MethylC CpHpG %	MethylC CpHpH %	Conv. rate %
Control 1	9,221,090	49	39	1	0	100
Control 2	14,000,485	28	39	1	1	99
Control 3	15,237,052	45	41	1	0	100
Control 4	16,305,850	45	48	1	1	99
Control 5	14,146,640	50	41	1	1	99
Control 6	17,961,183	26	39	0	0	100
Control 7	21,023,385	12	42	0	0	100
Control 8	14,287,174	45	40	1	1	99
Control 9	14,605,142	29	40	0	0	100
Control 10	19,983,522	49	50	1	1	99
Patient 1	20,739,258	25	42	0	0	100
Patient 2	12,830,866	51	38	1	1	99
Patient 3	16,521,910	34	43	1	1	99
Patient 4	16,888,563	30	39	1	1	99
Patient 5	13,589,812	51	42	0	0	100
Patient 6	14,374,956	49	43	0	0	100
Patient 7	21,391,108	40	39	1	0	100
Patient 8	13,587,009	37	41	1	1	99
Patient 9	13,261,182	28	61	1	1	99
Patient 10	20,052,690	46	45	1	1	99

Each sample, as identified by sample ID, has corresponding data showing, total reads, mapping efficiency from bismark, methylated cytosines in three different contexts (CpG, CpHpG and CpHpH). H can be either A, T or C, and the percentage is calculated individually for each context following the equation: % methylation = 100 × methylated Cs/(methylated Cs + unmethylated Cs). The bisulphite conversion rate calculated as the average of the number of Ts (non-methylated Cytosines) divided by coverage for each non-CpG cytosine

### Fragment-based differential DNA methylation pattern in ME/CFS patients:

**Fig. 2:**
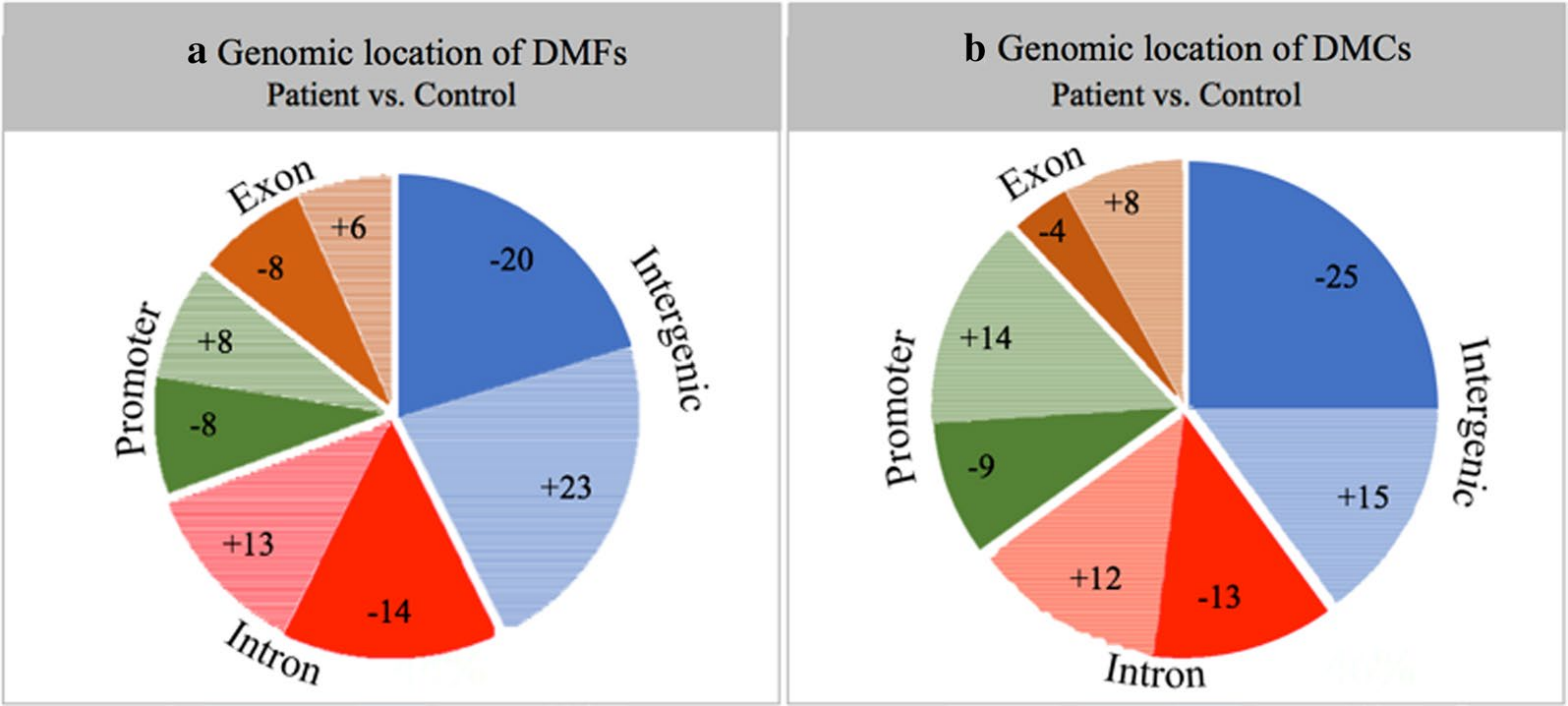
Pie charts showing proportional locations of differential methylation between patients and controls. **a** Genomic location of the differentially methylated fragments (DMFs) and **b** genomic location of the differentially methylated CpGs or DMCs***.*** The differential methylation was mapped to annotated human genome data including; (1) exons (brown), (2) intergenic regions (blue), (3) introns (red) and (4) promoter regions (defined as 1500 bp upstream and 500 bp downstream from the TSS) (green). Proportions of hypo-methylated sites/fragments are indicated by the darker ‘solid’ coloured segments, and hyper-methylated proportions by the lighter ‘shaded’ segments. ‘−’ is hypo-methylated, and ‘+’ is hyper-methylated. The percentages in each segment are shown

Using DMAP analysis, 76 DNA fragments were identified as differentially methylated out of 146,575 analysed RRBS fragments (with statistical thresholds set at a raw *P* value < 0.05 and a minimum methylation difference of 15%). These were named as differentially methylated fragments (DMFs). We have used a stringent *P* value threshold without false discovery rate correction in order to not lose true positives from this analysis, considering our sample size is low. The full list of DMFs is provided in Additional file 1: Excel file ‘DMAP_Diff_Fragments’. The 76 DMFs averaged 70 bp in length. Of these DMFs, 52% were hypo-methylated with 48% were hyper-methylated in ME/CFS patients compared with the healthy controls. The fragments were annotated and assigned to known genomic regions of interest (as shown in Fig. 2a). The major proportion of the DMFs were within intergenic regions (43%) and in intronic regions of protein-coding gene (27%) followed by gene promoters (16%) (defined as 500 bp downstream and 1500 bp upstream of the transcription start site), and exonic regions of genes (14%).

### Differential methylation pattern at single CpG sites in ME/CFS patients

Using the MethylKit pipeline, 349 from a total of 196,172 individual CpG sites were identified as differentially methylated CpGs (DMCs). They were determined to be statistically significant following a Fisher’s exact test with a FDR adjusted *P* value (*Q* value) of < 0.05 and a minimum methylation difference of 15% between the pooled control and patients’ samples (the full list of DMCs is available in Additional file 1: Excel file ‘MethylKit_Diff_Cytosines’).

Of the 349 DMCs, 56% were hypo-methylated and 44% hyper-methylated in ME/CFS patients compared with healthy controls. The sites were annotated to known genomic regions including promoter, intronic, exonic and intergenic regions (Fig. 2b). The highest proportion of DMCs fell within intergenic regions of the genome (40%) with the next largest falling within intronic regions (25%), followed by promoters (23%) (defined as 500 bp downstream and 1500 bp upstream of the transcription start site) and exons (12%). The distribution of differential methylation across genomic locations from both the MethylKit and DMAP analyses shows close concordance. This gives additional confidence to the results produced in the following analyses.

### Clusters of differential methylation within regulatory features identify mitochondrial and immune-related genes

**Fig. 3:**
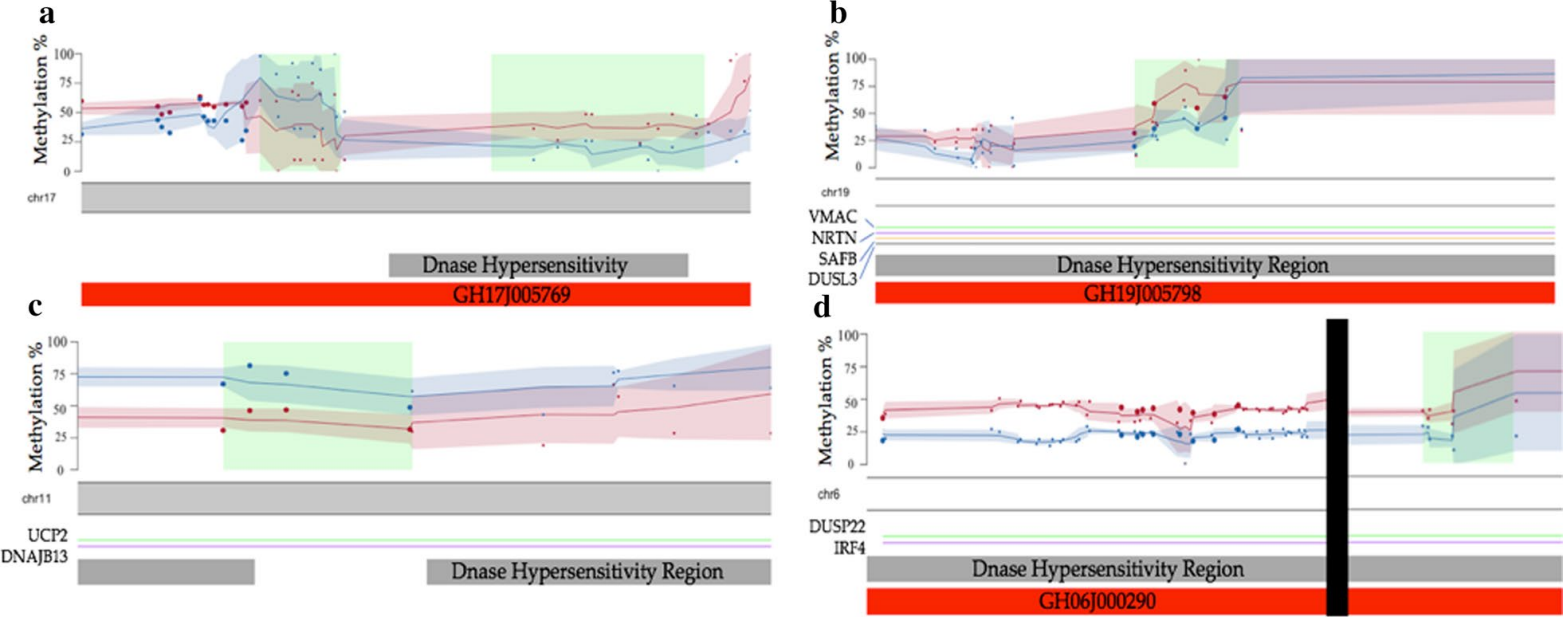
Clusters of Differential Methylation across four chromosomes. Average methylation percentages are shown at cytosines for patients (blue) and control (pink) samples. The pink and blue lines show the rolling mean methylation score across the fragment length with associated shaded area indicating standard deviation. Larger points indicate sites of differential methylation with a FDR rate corrected *Q* value < 0.05. Smaller dots sites of differential methylation level of significance > 0.05. Green blocks indicate the detected DMAP fragments with differential methylation. DNase hypersensitivity regions are shown in gray with enhancers shown in red. Regions of enhancer and gene interactions are shown with labels indicating the associated gene. **a** A 400 bp section of chromosome 17 shows differential methylation overlapping with a DNase hypersensitivity region, an Enhancer (GeneHancer ID: GH17J005769). **b** A section of chromosome 19 showing the differential methylation falling within DNase hypersensitivity cluster, an enhancer (GeneHancer ID: GH19J005798) and four regulatory interactions. **c** A section of chromosome 11 showing the closely clustered differential methylation overlapping a DNase hypersensitivity and two regulatory interactions. **d** 1570 bp section of chromosome 6 showing differential methylation overlapping with DNase hypersensitivity clusters, an enhancer (GeneHancer ID: GH06J000290) and two regulatory interactions. Note in D there is a zoomed view of the chromosome for the individual cytosine cluster (250 bp) and fragment (250 bp) (split by black vertical bar—representing 450 bp)

To determine sites of differential methylation linked to potential functional changes in ME/CFS, clusters were identified using the differential methylation profiles produced by both DMAP and MethylKit pipelines (see Fig. 3). Several overlapping clusters were identified from the MethylKit and DMAP analyses, indicating their importance within the genomic region at which they are located. For example, 13 DMCs found by MethylKit analysis were also found in the same genomic location as 7 DMFs using DMAP. There were an additional 24 DMCs identified from the Methylkit analysis in close proximity (< 2 Kbp up or downstream) to another 7 DMFs. Four clusters were identified with overlapping (Fig. 3b, c) or close proximity DMFs and 4 or more DMCs (Fig. 3a, d). Additional file 1: Excel file ‘Cluster_Data’ shows methylation scores at each cytosine and fragment within these four clusters.

The length of the genome covered by the clusters varied with the largest being 1570 bp and smallest 200 bp. Investigation of these regions of the genome revealed a number of regulatory features including enhancers, DNase hypersensitivity regions and regions of Enhancer/Gene regulatory associations recorded on the UCSC genome browser database (see Fig. 3). These regions of regulatory importance were associated with 17 protein-encoding genes with various functions, with the majority having strong links to mitochondrial function or the immune system (or both) (see Table 2).

**Table 2:** Genes linked to regulatory features overlapping with clusters of differential methylation

Gene ID	Regulatory element	Cluster	Description
XAF1	GH17J005769	CHR17	A negative regulator of the inhibitor of apoptosis (IAP) family resulting in increased stress induced apoptosis
ZNF594	GH17J005769	CHR17	Implicated in transcriptional regulation
DUSP22	GH06J000290 + Region of enhancer (GH06J000290) and gene interaction	CHR6	Activator of the JnK pathway involved in apoptosis, inflammation, cytokine production and metabolism
EXOC2	GH06J000290	CHR6	A component of the exocyst complex important for the targeting of vesicles to docking sites on the plasma membrane
IRF4	GH06J000290 + Region of enhancer (GH06J000225) and gene interaction	CHR6	A lymphocyte specific member of the interferon regulatory factor family of transcription factors for regulation of interferons in response to infection by virus
*UCP2*	*Region of enhancer (GH11J073778) and gene interaction*	*CHR11*	*A member of the mitochondrial uncoupling protein family that creates proton leaks across the inner mitochondrial membrane*
*DNAJB13*	*Region of enhancer (GH11J073968) and gene interaction*	*CHR11*	*A heat shock protein with an important role in the formation of the central complex of ciliary and flagellar axonemes*
DUS3L	Gene Body Overlap + GH19J005798 + Region of enhancer (GH19J006270) and gene interaction	CHR19	A protein catalyzing the synthesis of dihydrouridine
LONP1	GH19J005798	CHR19	A mitochondrial matrix protein that mediates the selective degradation of damage and the regulation of mitochondrial gene expression
CATSPERD	GH19J005798	CHR19	A component of the CatSper complex involved in sperm cell hyperactivation
FUT6	GH19J005798	CHR19	A Golgi stack membrane protein involved in the creation of the blood group antigen sialyl-Lewis X
FUT3	GH19J005798	CHR19	An enzyme that catalyzes the final step of Lewis antigen biosynthesis involved in the expression of; Vim-2, Lewis A, Lewis B, sialyl Lewis X and Lewix X/SSEA-1 antigens
FUT5	GH19J005798	CHR19	A paralog of FUT6
NDUFA11	GH19J005798	CHR19	A subunit of the membrane bound mitochondrial complex I which functions as the NADH-ubiquinol reductase of the mitochondrial electron transport chain
VMAC	GH19J005798 + Region of enhancer (GH19J005620) and gene interaction	CHR19	A vimentin-type intermediate filament-associated coiled-coil protein
NRTN	GH19J005798 + Region of enhancer (GH19J005798) and gene interaction	CHR19	A member of the GDNF family for survival and function of neurons. Implicated in immune responses with PBMCs—up-regulation indicating immune cells are communicating via NRTN
SAFB	Region of enhancer (GH19J006055) and gene interaction	CHR19	Implicated with stress responses, cell cycle, apoptosis and cell differentiation

Annotation includes the ‘GeneHancer’ ID of the associated differentially methylated enhancer, or the enhancer identified in the four clusters of differentially methylated region of regulatory interaction. Also included is the chromosome in which the cluster was located along with a brief description of the gene function. All are associated with hypo-methylated clusters except the rows highlighted italic that indicate association with a hyper-methylated cluster

Figure 3 shows rolling average methylation scores for the control and patients’ samples across DNA fragment lengths as indicated from chromosomes 6, 11, 17 and 19. Larger points indicate sites of differential methylation with a FDR rate corrected *Q* value < 0.05. Smaller points indicate observed differential methylation with a significance *Q* > 0.05. Annotation data were determined with the use of the UCSC Genome Browser with; DNase hypersensitivity regions, originally recorded in ENCODE from a collection of 125 different cell types, are shown in gray, with enhancers, registered in the GeneHancer database, are shown in red. Regions of enhancer and gene interactions are also shown with labels indicating the associated gene. Figure 3a shows a 400 bp section of chromosome 17 with differential methylation overlapping with a DNase hypersensitivity region, and an Enhancer (GeneHancer ID: GH17J005769). A section of chromosome 19 where the differential methylation falls within a DNase hypersensitivity cluster, an enhancer (GeneHancer ID: GH19J005798) and four regulatory interactions is shown in Fig. 3b. A section of chromosome 11 showing the closely clustered differential methylation overlapping a DNase hypersensitivity and two regulatory interactions is illustrated in Fig. 3c, d a 1570 bp section of chromosome 6 showing differential methylation overlapping with DNase hypersensitivity clusters, an enhancer (GeneHancer ID: GH06J000290) and two regulatory interactions.

## Investigating differential methylation within genomic features (gene bodies and promoter regions)

**Fig. 4:**
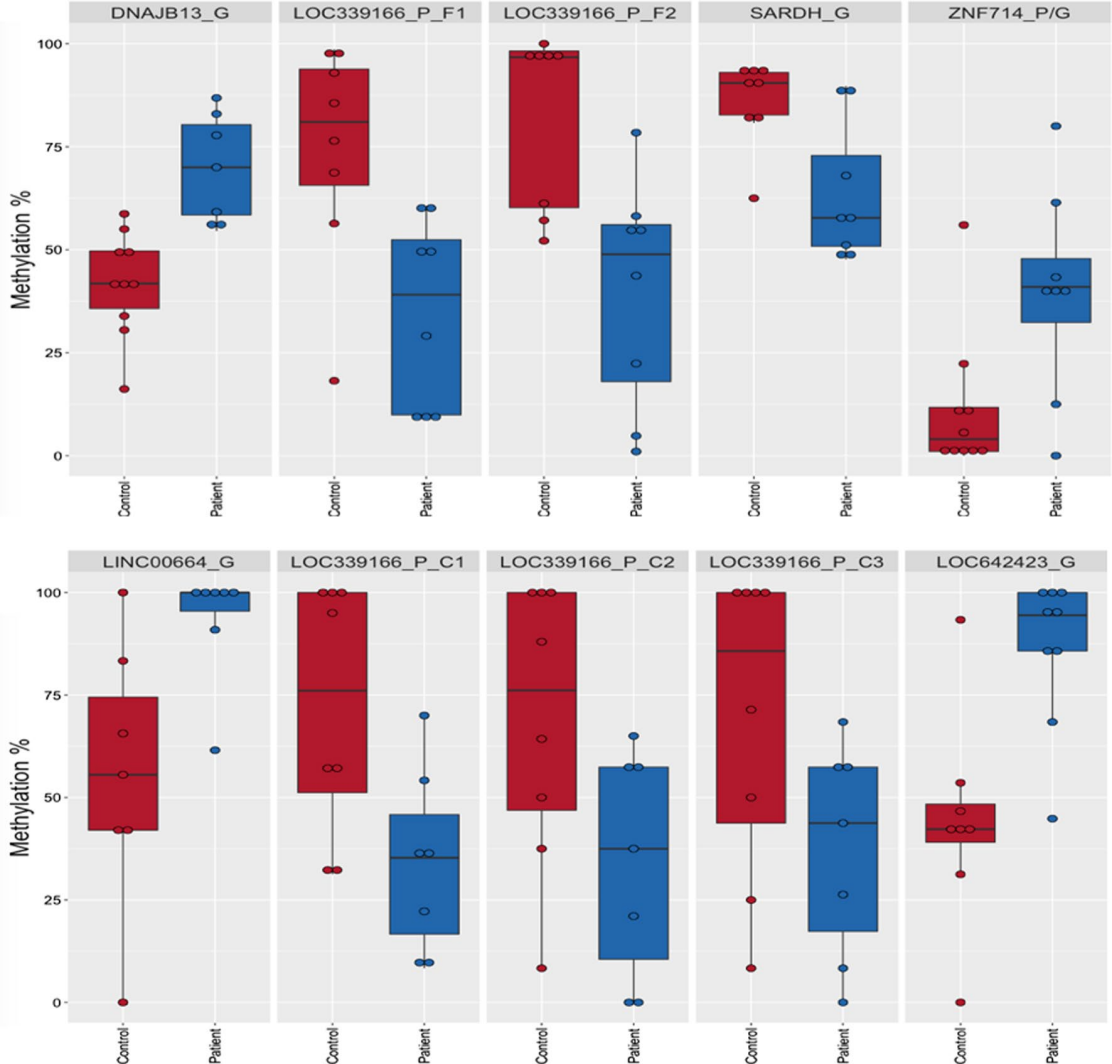
Differential methylation of potential key genomic features. Box plots showing the range of methylation percentages for the patients and controls with the range of the boxes indicating the limits of the upper third and lower third quartile of the data, with the mean indicated by the horizontal line within the box. Individual methylation percentages are shown as single data points. **a** The top five differentially methylated fragments within promoter or gene regions, and **b** the top differentially methylated individual cytosines within promoter or gene regions. Gene regions are indicated with a ‘G’ and promoter regions with a ‘P’ in the feature ID. Multiple cytosines from the same feature are indicated with a ‘C’ and an identifying number. Multiple fragments from the same feature are indicated with a ‘F’. Control boxes and points are shown in red with patients in blue

Investigating the location of individual DMCs and DMFs identified a number of genomic features of interest. Figure 4 shows the individual patient and control methylation values as individual points within the box plots for the top five differentially methylated (A) fragments and (B) individual cytosines within promoters and exon/intron genomic features. In the case of the LOC339166 promoter region, several sites were identified.

### Differential methylation within promoter regions of genes

Promoter regions were defined as being 1500 bp upstream and 500 bp downstream from the Transcription Start Site (TSS). Of the identified differentially methylated fragments (DMFs), 16% were found within these defined promoter regions (see Fig. 2a), and half were hypo-methylated and half hyper-methylated in ME/CFS patients compared to controls. Eleven different promoter regions were associated with twelve DMFs. The LOC339166 associated promoter region contained two adjacent statistically significant hypo-methylated fragments. The genes linked to promoter regions associated with the DMFs that show the most variation between the patients and controls are shown in Table 3, with values for both ME/CFS patients and healthy controls along with the *P* and *F* test values.

**Table 3:** Genes linked to promoter regions associated with the top DMFs

Gene ID	CpGs	Control %	Patient %	Difference	*P* value	*F *test value
LOC339166	11	75	33	− 42	6.85 × 10^–3^	*F*(1,14) = 10.03
LOC339166	8	80	46	− 34	3.78 × 10^–3^	*F*(1,14) = 12.02
ZNF876P	15	53	31	− 22	8.28 × 10^–3^	*F*(1,13) = 9.68
RAB20	5	42	25	− 17	5.20 × 10^–3^	*F*(1,16) = 10.46
NUDT14	3	38	21	− 17	5.30 × 10^–3^	*F*(1,14) = 10.87
MIR138-2	5	78	62	− 16	2.23 × 10^–2^	*F*(1,14) = 6.59
ZNF714	6	12	42	+ 30	1.12 × 10^–2^	*F*(1,16) = 8.22
C8orf31	3	43	64	+ 21	4.75 × 10^–2^	*F*(1,13) = 4.79
PAX8-AS1	7	65	83	+ 18	3.07 × 10^–2^	*F*(1,15) = 5.69
CCDC144NL	8	58	75	+ 17	3.36 × 10^–2^	*F*(1,16) = 5.41
LINC00923	4	60	76	+ 16	6.79 × 10^–3^	*F*(1,17) = 9.49
WNK2	17	38	54	+ 16	1.12 × 10^–2^	*F*(1,16) = 8.22

For each Gene ID, there is the corresponding number of CpGs within the identifying fragment, the average percentage methylation for the control subjects and ME/CFS patients, with differences (− is hypo-methylated, + is hyper-methylated). *P* values and* F* test values (degrees of freedom in brackets) are shown

The MethylKit analysis identified a larger number of promoter-associated genes with 45 DMCs falling within 22 promoter regions. Of these individual cytosines, 69% were hypo-methylated in ME/CFS patients compared to controls. There were two methylated promoter-associated genes that overlapped between the DMAP and MethylKit analyses, LOC339166 and NUDT14. The region associated with LOC339166 encompasses the cluster described previously within chromosome 17, and identifies regulatory interactions for XAF1 and ZNF594. NUDT14 is important for the elimination of toxic metabolites as well as the regulation of nucleotide substrates, cofactors and signalling molecules.

The genes linked to promoter regions associated with the top differentially methylated individual cytosines are listed in Table 4, with values shown for ME/CFS patients and healthy controls, along with their *P* and *Q* values.

**Table 4:** Genes linked to promoter regions associated with the top differentially methylated individual cytosines (5 of 31 hypo- and 5 of 14 hyper-methylated)

Gene ID	Control %	Patient %	Difference	*P* value	*Q *value
LOC339166	76	34	− 42	3.94 × 10^–12^	1.67 × 10^–9^
LOC339166	74	32	− 40	1.96 × 10^–11^	7.97 × 10^–9^
LOC339166	75	35	− 39	9.19 × 10^–11^	3.63 × 10^–8^
LOC339166	71	35	− 35	4.71 × 10^–9^	1.70 × 10^–6^
LOC339166	75	39	− 35	8.62 × 10^–9^	3.05 × 10^–6^
FANK1	19	48	+ 28	2.31 × 10^–12^	9.82 × 10^–10^
CILP2	30	51	+ 23	1.94 × 10^–4^	3.40 × 10^–2^
A2M-AS1	13	32	+ 21	4.93 × 10^–5^	1.05 × 10^–2^
SPIN2B	20	40	+ 20	2.45 × 10^–5^	5.58 × 10^–3^
MT1M	12	29	+ 18	1.21 × 10^–4^	2.29 × 10^–2^

For each Gene ID, there is the corresponding average percentage methylation for the control subjects and ME/CFS patients, with the differences shown (− is hypo-methylated, + is hyper-methylated). *P* and *Q* values are indicated. The full dataset is available in Additional file 1: Excel file ‘MethylKit_Promoter_Full’

### Differential methylation within gene bodies

A significant proportion of the DMFs determined with DMAP were located in intron and exon regions (26% and 14%, respectively—see Fig. 2). The DMAP fragment analysis identified 31 different genes within 31 fragments that had a total of 190 CpGs within them. The gene *GNG7* was associated with the fragment that had the most statistically significant differentially methylated CpGs. GNG7 is a guanine nucleotide-binding protein with a large range of functions including the regulation of adenylyl cyclase in the brain. Information concerning the length of the associated fragment, number of CpGs within the fragment, and patient, control and differential methylation percentages is shown in Table 5. Significance scores are shown as *P* values and a corresponding *F *value.

**Table 5:** Top hypo-methylated (5 out of 17) and top hyper-methylated (5 out of 14) methylated fragment-associated gene bodies (exons/introns)

Gene ID	CpGs	Control %	Patient %	Difference	*P* value	*F* test value
SARDH	3	88	64	− 24	6.52 × 10^–3^	*F*(1,14) = 10.19
MAST4	4	82	61	− 21	4.72 × 10^–3^	*F*(1,14) = 11.26
GRAMD4	4	64	44	− 20	4.80 × 10^–2^	*F*(1,13) = 4.76
GNG7	7	64	45	− 19	2.94 × 10^–2^	*F*(1,17) = 5.66
RP11-566K11.2	7	46	28	− 18	6.01 × 10^–2^	*F*(1,15) = 18.70
ZNF714	6	12	42	+ 30	1.12 × 10^–2^	*F*(1,16) = 8.22
DNAJB13	5	41	69	+ 28	4.57 × 10^–4^	*F*(1,15) = 19.91
COX19	6	11	32	+ 21	1.96 × 10^–2^	*F*(1,15) = 6.82
CARD8	3	77	98	+ 21	7.06 × 10^–4^	*F*(1,13) = 19.44
C8orf31	9	43	64	+ 21	4.75 × 10^–2^	*F*(1,13) = 4.79

For each gene, the number of CpGs within the fragment, and methylation percentages of control subjects and ME/CFS patients are shown, with the differences (− is hypo-methylated, + is hyper-methylated). Significance scores are shown as *P* values and corresponding *F* value (degrees of freedom in brackets). A full list is available in Additional file 1: Excel file ‘DMAP_Gene_Full’.

Additionally, a total of 121 DMCs were found in 91 different genes identified through the MethylKit analysis. There were equal proportions of hyper- and hypo-methylated sites in ME/CFS patients compared with healthy controls within these gene regions (51% and 49%, respectively). The protein-encoding genes with the most DMCs (MethylKit analysis) were *PARD6* with 6 hypo-methylated cytosines and *SKA3* with 6 hyper-methylated cytosines. Both have roles in cell division. There were no overlaps between the separate DMAP and MethylKit analyses in the gene bodies they identified. Table 6 shows the top 5 hypo- and hyper-methylated genes in patients compared to controls including the separate control and patient methylation percentages and corresponding significance values.

**Table 6:** Top hypo-methylated (5 of 43) and hyper-methylated (5 of 41) individual cytosines associated with gene bodies (exons/introns)

Gene ID	Control %	Patient %	Difference	*P* value	*Q *value
MEGF6	55	17	− 38	1.12 × 10^–11^	4.63 × 10^–9^
CD6	41	3	− 38	1.90 × 10^–16^	1.02 × 10^–13^
KIF1A	50	14	− 36	1.83 × 10^–10^	7.16 × 10^–8^
MEGF11	84	52	− 32	6.09 × 10^–12^	2.54 × 10^–9^
TMCO3	94	61	− 33	3.28 × 10^–9^	1.20 × 10^–6^
LINC00664	53	95	+ 42	2.95 × 10^–15^	1.50 × 10^–12^
LOC642423	43	84	+ 41	2.60 × 10^–14^	1.24 × 10^–11^
KCNK10	54	93	+ 39	2.92 × 10^–17^	1.60 × 10^–14^
IDUA	59	95	+ 36	1.21 × 10^–13^	5.58 × 10^–11^
FAM182B	0	34	+ 34	8.99 × 10^–16^	4.71 × 10^–13^

Gene-associated cytosine significance values were calculated using a Fisher’s test. For each cytosine, there is also corresponding genomic location, the average methylation percentages for the control subjects and ME/CFS patients, with differences (− hypo-methylated, + is hyper-methylated). Corresponding *P* and *Q* values are indicated. The full dataset is available in Additional file 1: Excel file ‘MethylKit_Gene_Full’

Tables 3 and 4 list the differentially methylated genes linked to promoter regions identified, and Tables 5 and 6 the differentially methylated gene bodies with the two analysis platforms.

### Validation of RRBS methylomes of ME/CFS patients with independent cohorts from published array based methylome studies

Till date, five published studies have compared the methylation states of cohorts of ME/CFS patients compared with healthy matched controls [6–10]. All these studies have utilised array-based methods with either the Infinium HumanMethylation450 BeadChip [6–8, 10] or the Illumina Methylation EPIC microarray [9]. The array-based platforms by design covers less number of CpG sites. Our study, utilising reduced representation bisulphite sequencing (RRBS), is the first study with ME/CFS patients to use this method—has identified differential methylation across the genome in regions enriched with functional CpG sites. Extensive validation of this method and the analytical platforms has been carried out previously by our group and others [19–21], and RRBS was also used to generate reproducible methylomes in multiple organisms as well (human, mouse, zebrafish) [22–24]. While the two methods produce very different raw outputs, it is still possible to compare the overlaps in the processed data such as was performed below where comparisons of the differentially methylated gene lists were investigated to independently validate our RRBS results.

**Fig. 5:**
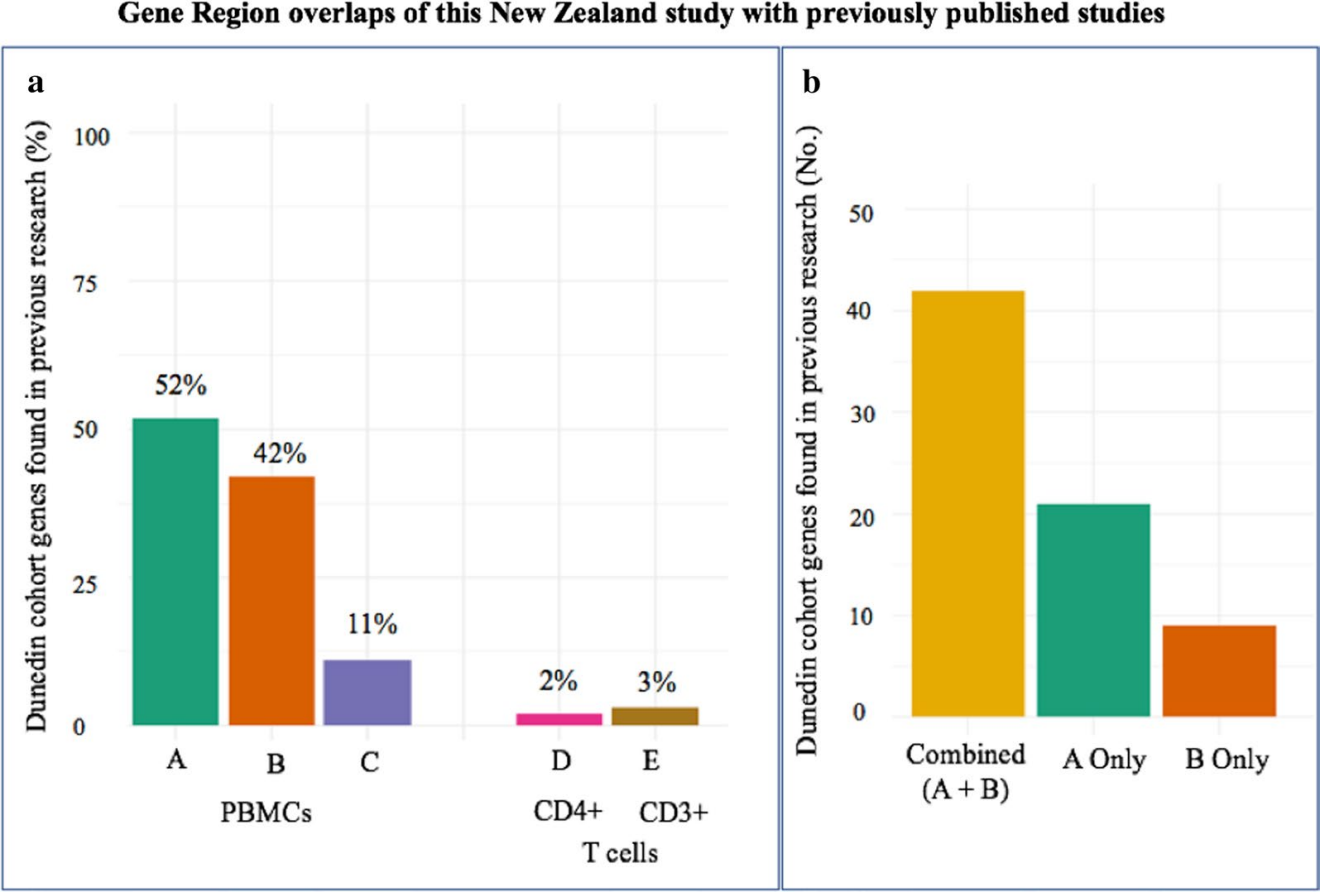
Overlaps observed between the genes identified in this New Zealand study and previously published studies. **a** Bar plot showing the percentage of genes identified in the New Zealand study described here that overlap with previous published work that assessed the methylome of ME/CFS patients compared to healthy controls. **a** Bar A is the Trivedi et al. 2018 study [9], bar B is de Vega et al. [8], bar C is de Vega et al. [7], bar D is Brenu et al. [6], and bar E is Herrera et al. [10]. A summary of the genes found to be overlapping with each study is provided in Additional file 1: Excel file ‘Genelist_Overlaps’. **b** Bar plots showing the number of the 122 genes identified in the New Zealand study that overlapped with: ‘(A + B)’—*both* the Trivedi et al. [9] and de Vega et al. [8] studies; ‘A only’—Trivedi et al. [9], ‘B only’—de Vega et al. [8]. None of the overlaps between our New Zealand study and the de Vega et al. [7] (bar C in Fig. 5a) were unique to that study, but were also found in the other two studies [8, 9]

Figure 5a shows that those genes differentially methylated in our study were also identified in each of the other studies. A list of the specific genes in common with each of those studies is shown in Additional file 1: Excel file ‘Genelist_Overlaps’. Three studies also used PBMC cells (a mixture of monocytes and lymphocytes) as in our study, (1) de Vega et al. [7], (2) de Vega et al. [8] and (3) Trivedi et al. [9]. The other two studies targeted specific T cell subpopulations, CD4 + T cells [6], and CD3 + T cells [10]. Of the 122 genes found in our New Zealand study, 52% were also identified in the Trivedi et al. 2018 study [(A) in Fig. 5a], and 42% in the 2017 de Vega et al. study [B in Fig. 5a]. A smaller study in 2014 by de Vega et al. [C in Fig. 5a] had identified 11% of the genes that were found in the current study. By contrast, the other two studies with the specific cell subtypes had only a small amount of overlap (2% and 3%) (D and E in Fig. 5a).

We were interested also to determine whether genes identified in the current study were found in both the Trivedi et al. and de Vega et al. studies. As shown in Fig. 5b, of the 122 genes identified by the two analysis platforms in our study, 42 were identified in all 3 studies (A + B + New Zealand), 21 in only Trivedi et al. 2018 and our study (A + New Zealand), and 9 only de Vega et al. 2017 and our study (B + New Zealand). Hence 72/122 genes identified in our study have been identified in the two studies with significant overlap. Genes (13/14) overlapping with the smaller de Vega et al. 2014 study were also found in the other two studies (that is all 4 studies), and (1/14) with the Trivedi et al. 2018 study only (that is, in 3 studies). These results strongly validates our RRBS results with an independent platform and cohorts.

### Do identified differentially methylated DNA fragments and differentially methylated individual cytosines relate to ME/CFS pathophysiology?

To determine whether the genomic elements associated with the DMFs and individual DMCs were identifying systemic changes relevant to ME/CFS pathophysiology, a functional enrichment analysis was performed through STRING.org v.11.0 [25]. No significant enriched functional pathways were identified based on the differentially methylated promoter-associated genes through either the DMAP or MethylKit analysis.

**Fig. 6:**
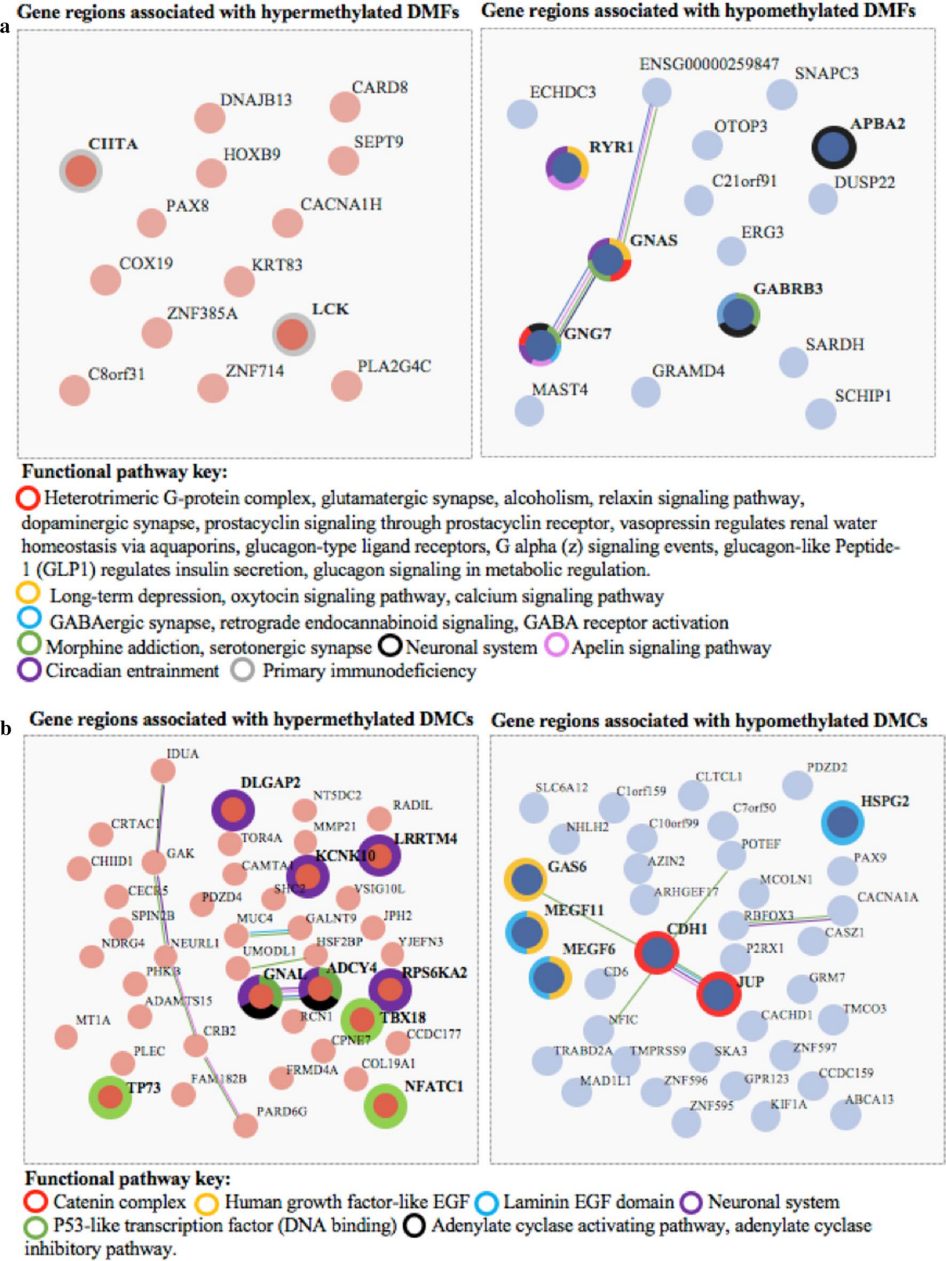
STRING diagrams showing functional relationships between hyper- and hypo-methylated DMFs within gene regions (**a**) and functional relationships between hyper- and hypo-methylated DMCs within gene regions (**b**). Colours highlighting specific genes indicate their presence within an overrepresented functional pathway determined through a STRING analysis. Functional pathways all have a FDR-corrected *P* value < 0.05. A full list of the functional pathways with associated *P* values and gene numbers in sets is included in Additional file 1: Excel file ‘DMAP_Pathways and ‘MethylKit_Pathways’

Functional analysis was performed on the 31 genes associated with DMFs identified in the DMAP analysis and the 91 genes associated with the DMC analysis. Both STRING analyses were performed with the FDR *P* value cut-off of < 0.05. The enrichment analysis on the gene bodies associated with hyper- and hypo-methylated fragments identified in the DMAP analysis revealed a total of 22 functional pathways, 21 associated with hypo-methylated gene bodies and 1 associated with hyper-methylated gene bodies (Fig. 6a). From the 31 genes in the analysis, the enriched functional pathways associated with hypo-methylation were identified by various combinations of the following five genes: *RYR1,* *GNAS,* *GNG7,* *GABRB3* and *APBA3*, and the single hyper-methylation-associated functional pathway was identified with two hyper-methylated genes: *LCK* and *CIITA*.

In Fig. 6a, the gene bodies (exon/intron) of specific genes associated with DMFs are illustrated with hypo-methylated (pink) and hyper-methylated (blue). Represented in the halos surrounding the gene body are the pathway(s) associated with that gene (colours indicated in the key below A). For example, GNG7 (right panel) is involved in multiple functional pathways including: heterotrimeric complex, neuronal system, serotonergic synapse, apelin signalling pathway and circadian entrainment. In Fig. 6b, similar patterns are shown for the gene regions (exon/intron) derived from the DMCs, with the colours of the halo illustrating the associated pathways. The lines in the diagrams show STRING predicted associations. A total of 7 pathways were associated with the 91 differentially methylated gene bodies (exons/introns) identified from the single cytosine Methylkit analysis; 4 of those were from hyper-methylated sites, and 3 from hypo-methylated sites (Fig. 6b).

## Discussion

### The first study with reduced representation bisulphite sequencing

This is the first study of its kind to explore the reduced DNA methylome of ME/CFS patient PBMCs utilising reduced representation bisulphite sequencing (RRBS). This approach has identified a number of differentially methylated genomic features through multiple statistical analyses. These features were associated with a number of specific functions as well as enriched functional pathways. The data indicated an imprint of a systemic disruption revealed in the changed methylome of ME/CFS patients, particularly implicating immune, energy metabolism and neurological disruptions.

Since its first use in 2005, RRBS has been utilised by a number of international research groups producing reliable and reproducible results in a number of large and varied investigations across different tissue types and species [26–28]. The reliability of RRBS is strongly supported by work performed utilising the same RRBS platform as in this analysis that has been well validated in prior investigations [20, 21]. Chatterjee et al. 2016 demonstrated that the platform used for this investigation is highly reliable with replicate RRBS library preparations sequenced on different flow cells producing very consistent outputs (Pearson’s correlation coefficient of 0.98) [20]. The RRBS platform has also been validated through additional work performed previously [21]. The reliability of RRBS-based data was validated against other small-scale sequencing platforms through comparison with the Sequenom EpiTyper platform. A correlation (Pearson’s correlation coefficient) of 0.98 was observed when fragment methylation values were compared across four genes [21]. This provides confidence in the reliability of the technical validity of the method and the results of the current study.

Multiple statistical approaches were taken in order to detect as many meaningful changes as possible. In order to detect broad changes in DNA methylation likely linked to functional changes, the fragment-based DMAP analysis was utilised. To both support and build upon this initial approach, individual cytosine methylation was also interrogated utilising MethylKit. As would be expected both showed similar overall distributions of differential methylation (Fig. 2) in addition to a small number of shared features identified in the most differentially methylated DMFs and individual cytosines. However, there were also many differences in the outputs between the two analyses of the promoter region and of the gene bodies (exons/introns), and consequent enrichment analysis highlighting the divergence of results that can occur when utilising different statistical approaches.

This is especially relevant for making comparisons among the studies that have investigated DNA methylation changes in ME/CFS cohorts where varying results were obtained [6–10]. Among them there are differences in study design with consideration of (1) clinical case definition used for diagnosis, (2) methodology, (3) study population and (4) statistical analysis. Table 7 summarises the design variations and outputs in the published work and our study. There are differences in both study designs and analysis strategies in the studies that utilised array-based platforms. The variations seen in the tissue type utilised, cohort designs and particularly the differences in the statistical thresholds set in the analyses have, not surprisingly, produced variation in the outcomes. This is seen in the number of statistically significant differentially methylated sites observed by each study and the surprisingly wide divergence even in the overall proportions of hypo- and hyper- methylation (Table 7). Two studies report almost exclusively hypo-methylation changes [6, 9], while the other three predominately hyper-methylated changes [7, 8, 10]. In our NZ study, there was a more even distribution between hypo-methylated, and hyper-methylated sites, but with differentially methylated cytosines within the promoter regions predominantly hypo-methylated (~ 70%) and, as discussed below, the majority of the functional pathways identified were due to the presence of five *hypo*-methylated genes.

**Table 7:** Comparisons of study features investigating DNA methylation across the genome of ME/CFS patients compared to controls

Study	Tissue	Method	Cohort	Diagnostic criteria	Statistical thresholds	No. of significant differences
NZ	PBMCs	Reduced representation bisulphite sequencing	*P* = 10 5 females 5 males *C* = 10 5 females 5 males	Canadian criteria	DMAP ANOVA F test Raw *P* < 0.05, methylation diff ± 15% MethylKit Fisher’s exact test FDR corrected *P* < 0.05. methylation diff ± 15%	DMAP: 76 (52% hypo-methylated) Methylkit: 394 (56% hypo-methylated)
A	CD4 + T cells	Infinium HumanMethylation450 BeadChip	*P* = 25 21 females 4 males *C* = 18 10 females 8 males	Fukuda criteria	Raw *P* value < 0.05, methylation fold change > 2.0	120 (85% hypo-methylated)
B	PBMCs	Infinium HumanMethylation450 BeadChip	*P* = 12, *C* = 12 All female	Fukuda and Canadian criteria	Wilcoxon-rank sum test *P* < 0.05, FDR corrected *P* < 0.05 (Benjamini-Hochberg). Mean beta difference > 0.20	1192 (72% hyper-methylated)
C	PBMCs	Infinium HumanMethylation450 BeadChip	*P* = 49, *C* = 25 All female	Fukuda and Canadian criteria	Wilcoxon-rank sum test *P* < 0.05, FDR corrected *P* < 0.05. Mean beta difference > 0.05	12,608 (71.6% hyper-methylated)
D*	PBMCs	Illumina Methylation EPIC microarray	*P* = 13, *C* = 12 All female	Fukuda and Canadian criteria	FDR-corrected *P* value < 0.05 absolute beta difference > 0.05	17,296 (98% hypo-methylated)
E	CD3 + T Cells	Infinium HumanMethylation450 BeadChip	*P* = 43 34 females 9 males *C* = 36 27 females 9 males)	Fukuda and Canadian criteria	*P* value < 0.05 permutation analysis, mean percentage methylation difference > 5%	133 (74% hyper-methylated)

A table comparing the NZ study with the five previous studies investigating DNA methylation in ME/CFS patients vs. controls. (NZ = this study, A = Brenu et al. 2014 [6], B = de Vega et al. 2014 [7], *C* = de Vega et al. 2017 [8], D = Trivedi et al. 2018 [9], E = Herrera et al. 2018 [10]) The table includes the cell type utilised, the method used in the analysis, the numbers and gender included in the cohort, diagnostic criteria of the patients and the statistical thresholds utilised in the analyses. *D (Trivedi et al. [9]) included a larger cohort for pyrosequencing validation with a total of 33 cases and 31 controls from three geographical locations

The NZ study used the Canadian Consensus Criteria [29] for diagnosis, while the other studies used patients diagnosed by this criteria and the earlier 1994 Fukuda diagnostic criteria [30] developed by the Centre for Disease Control in the USA. While this has been most commonly used by researchers and clinicians [31], it does not include the core defining symptoms of post-exertional malaise and neurocognitive disturbances, nor does it exclude patients whose symptoms may originate from a psychiatric disorder. The Canadian Consensus Criteria (CCC) [29] was developed in 2003 by an international ME/CFS expert group, highlighting post-exertional malaise as a core symptom, along with fatigue, sleep dysfunction and pain. Additionally, neurological/cognitive and autonomic/neuroendocrine/immune symptom groups were included. The NZ study like Herrera et al. [10] and Brenu et al. [6] had both male and female subjects, while the other studies were exclusively female. The statistical thresholds set for the analyses can result in variations among the published studies in the identification of differentially methylated sites, for example, utilising uncorrected *P* values < 0.001 [6] compared to the more stringent FDR corrected *P* values < 0.05 [7–9] that remove potential false positives from the data. Another important distinction is the variation in the threshold set for ‘significant’ methylation differences across the studies. The previous studies utilised array-based technologies, so methylation differences were calculated as beta differences, i.e. the ratio of probe intensity over total intensity used as an estimate of methylation percentage at each probe. The ‘significant methylation differences’ required between the patient and control groups varied across the studies from a methylation (β value) fold change > 2.0 [6] to a beta difference of > 0.2 [7] and > 0.05 [8–9]. This means the number of statistically significant differentially methylated sites identified by each of these studies and the present study would be expected to be different according to the different statistical criteria set to identify significant changes between the ME/CFS patients and controls (see Table 7).

### Results of the NZ study in comparison with the published studies

We compared the available gene lists produced by the array-based analyses [6–10] performed in these five previous investigations with those derived from our New Zealand study using RRBS. This revealed that 59% (72/122) of the genes identified in the New Zealand study had been observed in one or more of the previous studies, with 34% (42/122) observed in two comparable studies using PBMCs [8–9]. This indicates that even with ME/CFS cohorts diagnosed by different criteria and differing in age range, gender, stage of illness, nationality, and with significant variations in investigative processes, it has been possible to detect specific ME/CFS differential methylation compared with healthy controls. Interestingly, the two studies utilising sub populations of T cells showed only small overlaps in the genes showing differential methylation suggesting the changes are specific to the particular physiological functions of those cells in the ME/CFS illness.

### DNA methylation changes in the regulatory regions of genes

As DNA methylation within the regulatory regions of genes has been known to negatively correlate with gene expression, identification of differential methylation within these regions in our study has potential functional implications for ME/CFS patients compared to healthy controls. Four clusters of differential methylation, identified using both DMAP and MethylKit data, were observed across regions of the genome with a number of regulatory interactions. Two primary functional groupings, mitochondrial and immune, were observed across the genes associated with these differentially methylated regulatory regions, supporting an association between the observed differential methylation patterns and the pathophysiology of ME/CFS.

Clusters of differential methylation in the regulatory regions of genes UCP2, LONP1 and NDUFA11 related to mitochondrial function identified in this study suggest impaired functioning. UCP2 is an uncoupling protein capable of dissipating the proton gradient generated by NADH-powered pumping of protons into the inter-membrane space with associated links to energy metabolism efficiency [32]. It has been positively associated with reactive oxygen species (ROS) and reduces their generation making it important for cellular protection. As the enhancer for this gene was *hyper-*methylated, it indicates ME/CFS patients may exist in a state of impaired energy metabolism in addition to being more vulnerable to ROS. Additionally, LONP1 is a stress-associated protein involved in the maintenance and function of the mitochondria by removing oxidised proteins from the mitochondrial matrix. Increased levels are associated with acute stress [33]. As the enhancer region was *hypo*-methylated, it is likely that ME/CFS patients have an overexpression of LONP1 compared to healthy controls providing an explanation why they exist in an irregular state of acute stress, well documented clinically. NDUFA11 is a subunit of the membrane bound mitochondrial complex 1 (NADH dehydrogenase) [34] and with the enhancer region *hypo*-methylated in ME/CFS patients an associated increase in expression may be indicative of a compensatory response to an overall mitochondrial dysfunction, as has been suggested by Missailidis et al. [5].

Mitochondrial function and energy metabolism have previously been shown to be impaired in other approaches in ME/CFS patients and, while specific findings from study to study are inconsistent [35–38]. Previous work from our research group on the NZ cohort of ME/CFS patients characterised the PBMC transcriptome [39] identifying a significant increase in expression of a component of the first enzyme complex in the electron transport chain—NDUFS6, also a subunit of the NADH dehydrogenase. Additionally, to gain further insight into the pathophysiology of ME/CFS, we have very recently assessed the proteomes of PBMCs by SWATH-MS analysis in our small well-characterised group of patients and matched controls [40]. A total of 60 proteins in the ME/CFS patients were differentially expressed (*P* < 0.01, Log10 (Fold Change) > 0.2 and < − 0.2). A proportion of the identified proteins in the ME/CFS groups were involved in mitochondrial function, oxidative phosphorylation, electron transport chain complexes, and redox regulation. A significant number were also involved in previously implicated disturbances in ME/CFS, such as the immune and inflammatory response, DNA methylation, apoptosis and proteasome activation [40]. The results from this study also support a model of deficient ATP production in ME/CFS, compensated for by upregulation of pathways immediately upstream of Complex V that would suggest an elevation of oxidative stress. Missailidis et al. [5] concluded the *activity* of mitochondrial complex V involved in the actual synthesis of ATP is impaired resulting in up-regulation of remaining complexes and in particular complex 1 resulting in an inability to compensate for energy need during activity and stress. A predicted increase in the expression of the first mitochondrial electron chain complex in this epigenetic study and our previous studies and that of Missailidis et al. implicates an attempt to compensate for impaired energy production in these cohorts of ME/CFS patients.

The observed differential methylation in regulatory regions of genes associated with immune and inflammatory responses is not unexpected due the known pathophysiology of ME/CFS. Prior work has identified abnormalities in cytokine profiles [41] with additional supporting work indicating abnormal T lymphocyte activation and impaired cytotoxic responses [42]. The combination of these observations and the prolonged flu-like symptoms of ME/CFS indicates that an immune impairment is a key component of the disease. Nine genes associated with clusters of differential methylation identified in this study have roles with links to immune function and inflammation such as the gene product IRF4; an interferon regulatory factor important for the protection of the cell from viral infection through the activation of immune responses. EXOC2 is also involved in immune responses against viral infection through the co-localisation with STING and subsequent stimulation of interferon genes [43]. The hypo-methylation of the *IRF4* and *EXOC2* associated enhancer regions in ME/CFS patients indicates a potential up-regulation of their associated proteins. As increased interferon presence in patients has already been hypothesised to contribute to the prolonged fatigue [44], this up-regulation of the interferon response may reflect symptoms of fever, muscle pain and flu-like symptoms found in ME/CFS patients. 

A number of the previous investigations into the methylome of ME/CFS patients have highlighted immune activity as being potentially dysfunctional based on the outcomes of their analyses [6–7, 9]. Earlier work identified the immune KEGG pathway ‘Antigen processing and presentation’ [6] as being significantly enriched through the genes associated with differentially methylated probes, and another study identified an over-representation of GO terms related to immune cell regulation [7] indicating widespread irregular immune activity. Additional research identified gene pathway enrichment of a number of immune-regulatory stress response pathways including the p38 MAPK pathway which is involved in the response to inflammatory cytokines. Furthermore, they also identified a number of enriched interleukin signalling pathways including IL-8 [9]. IL8 had been identified as the gene with the most enhanced expression in our transcriptome analysis on the NZ cohort in ME/CFS patients compared to controls [39].

DNA methylation within gene bodies (exons/introns) has also been associated with changes in gene expression with hyper-methylation of the first exon often associated with an inverse correlation with gene expression (11). However, internal intron and exon methylation has also been observed with a positive correlation with gene expression (45). In order to explore the systematic changes in expression, pathway enrichment analysis of the genes associated with differential methylation was performed and identified a large number of functional pathways with links to the known clinical presentation of ME/CFS.

The KEGG pathway ‘Circadian entrainment’ was identified in this analysis through genes associated with *hypo*-methylation within internal introns. This is potentially indicative of an irregular circadian rhythm being established in ME/CFS patients vs. healthy controls, especially with the hypo-methylated ryanodine receptor RYR1 having an important role setting the clock to the evening phase in response to light [46]. Our previous transcriptome study performed on the same NZ cohort of patients observed changes in the expression of genes encoding circadian rhythm-related proteins, indicating a disruption to circadian regulation [38]. The correlations across these biological investigations with patient symptoms are providing a significant crossover with the overall clinical presentation of ME/CFS, with symptoms such as fatigue, sleep and cognitive dysfunctions, flu-like symptoms and metabolic and immune disruptions all having links to disrupted circadian activity.

The majority of the functional pathways identified were due to the presence of five *hypo*-methylated genes (internal intron); *RYR1,* *GNAS,* *GNG7,* *GABRB3* and *APBA2,* which in various combinations identified 22 enriched functional pathways in the analysis. It is interesting to note that the gene bodies of *RYR1*, *GNG7,* *GNAS* and *APBA2* have been associated with differentially methylated probes in multiple previous publications (for example 8, 9) (see Additional file 1: Excel file ‘Gene_Overlaps’) described in the results section. *RYR1* is a ryanodine receptor that acts as a calcium release channel within skeletal muscle. It is also involved in the establishment of circadian rhythms as it is known to release calcium in response to light, synchronising the internal biological clock—the suprachiasmatic nucleus [46]. Both *GNAS* and *GNG7* are guanine nucleotide-binding protein subunits important for the activity of transmembrane signalling systems in particular the adenylyl cyclase signalling activity and subsequently cAMP [47, 48]. *GABRB3* is a receptor for gamma-aminobutyric acid (GABA) and is therefore plays an important role in the function of the inhibitory neurotransmitter (GABA) within the central nervous system [49] *APBA2* is a neuronal adaptor protein essential for neurotransmitter release [50]. These specific functions provide strong validation to the biological relevance of the observations here highlighting the importance of the functional pathways to the pathophysiology of ME/CFS. Of these pathways, a large number were neurotransmitter and neuropeptide-related KEGG Pathways including; Serotonergic, Glutamatergic, GABAergic, Oxytocin, Dopaminergic, Apelin and Relaxin signalling. Neurotransmitter activity such as that of 5-methoxytryptamine (5-MT) (a compound critical to serotonin and melatonin metabolism) and neurotransmitter metabolites has been correlated with ME/CFS symptom severity previously indicating that these overrepresented pathways are potentially informative of ME/CFS pathophysiology [51]. Neurotransmitters themselves have been hypothesised to play a role in the symptom presentation of ME/CFS with excess levels of serotonin known to have impacts on an individual’s cognition, neuromuscular activity and autonomic system [52].

**Fig. 7:**
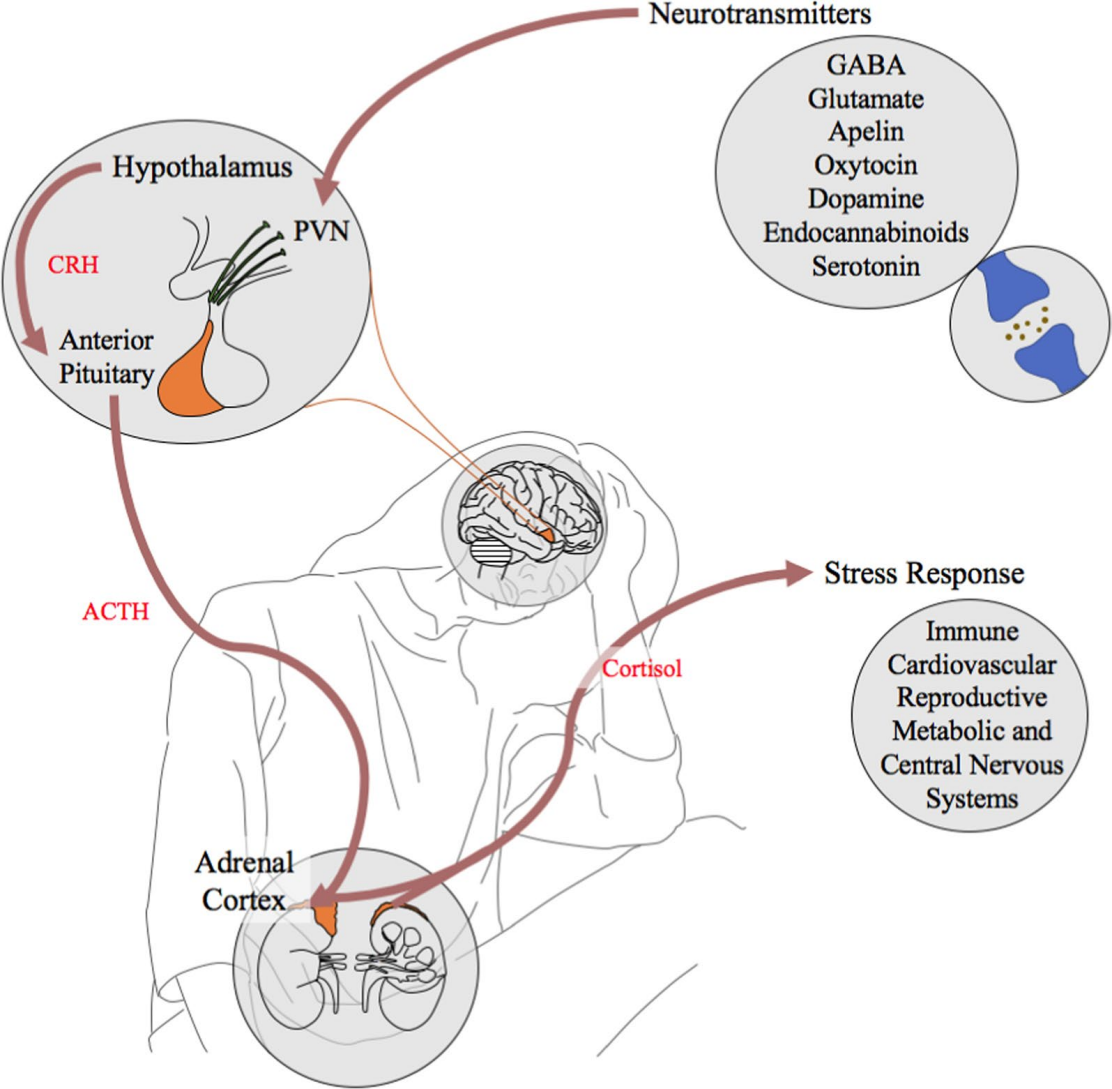
The HPA axis including the overrepresented neurotransmitter pathways in ME/CFS identified by this analysis. The pathways identified from the overrepresentation analysis and shown here are known to stimulate the HPA axis either directly through the paraventricular nucleus (PVN) with corticotropin releasing hormone (CRH)-producing cells or by an unknown mechanism linked to it. The activation of the CRH sensitive neuronal cells then triggers a downstream stimulation of the anterior pituitary causing the release of adrenocorticotropic hormone (ACTH). ACTH stimulates the adrenal cortex releasing glucocorticoids including cortisol into the body. Cortisol then has a role in the stimulation of a large number of downstream systems involved in a stress response

Together the combination of neurotransmitter functional pathways identified in this analysis provides a compelling addition to the hypothesis indicating that a dysfunctional HPA axis of ME/CFS patients is at least in part responsible for their prolonged disease state [53]. Many previous studies have focused on the link between epigenetic irregularities and the HPA axis activity in ME/CFS pathophysiology with the most compelling outcome the identification of 13 differentially methylated sites associated with glucocorticoid hypersensitivity in ME/CFS patients [8]. The HPA axis is stimulated by the paraventricular nucleus (PVN) within it. This structure contains a cluster of neurons regulating stress responses, and it is hypothesised to be a critical core centre for the ME/CFS condition, supporting prolonged and fluctuating disease [54]. The PVN is regulated itself by a number of neurotransmitters including those identified in this work such as apelin, serotonin, glutamate, GABA, endocannabinoids and dopamine. Figure 7 presents a summary of the HPA axis, and how our study of the predicted overexpressed neurotransmitters shown in the figure can invoke a stress response affecting many important physiological systems in ME/CFS.

The HPA axis is involved in a variety of functions from circadian regulation to the stimulation of stress responses to external stimuli. It impacts on cardiovascular, reproductive, metabolic, immune and nervous systems, and its dysfunction is connected to the clinical presentation of ME/CFS providing a compelling yet broad target for further research.

Our study demonstrates how DNA methylation has provided an imprint of multiple systemic changes in ME/CFS with links to disease pathophysiology. Comparisons with previous relevant publications have provided compelling support that the genes identified in this work are reflecting changes specific to an ME/CFS state. Many of the specific targets highlighted can now become the focus of validation and stimulation of further work to ameliorate the devastating effects of ME/CFS on those affected by the disease.

## Methods

### Cohort recruitment

ME/CFS patients and controls were recruited from Dunedin NZ. Diagnosis of ME/CFS was made by Dr Rosamund Vallings of the Howick Health and Medical Centre, Auckland, NZ, using the Canadian consensus diagnostic criteria [29]. Patients and controls were age- and sex-matched. Patients completed a questionnaire at the onset of this study, a blank copy of which is included as Additional file 3: Word document: ‘Questionnaire’. They ranged in age from 9 to 80 and included both males and females, with an illness duration averaging 12.5 years. Seven of the ten patients had a sudden onset with the remaining three reporting either a period of 1–12 months to over 12 months for onset. All patients report a loss of normal function with the majority either housebound or with limited activity and unable to work. Only two patients are able to work in any capacity (part time). Patients included in the cohort reported frequent suffering of symptoms, in particular; ‘persistent fatigue’, ‘exhaustion from physical or mental activity’, ‘physical/mental activity resulting in worsening of symptoms’, ‘intolerance to stress’ and notably ‘recovery after activity taking longer after their ME/CFS onset’. The majority of patients reported to be affected either ‘severely’ or ‘very severely’ for these symptoms. Additional details of the cohort can be found in Additional file 1: Excel file ‘Patient_Background’ and ‘Symptom_Presentation’, and Additional file 2: Figure S1. The study conforms to the ethics approval 17/STH/188 for ME/CFS patient studies, from the Southern Health and Disability Ethics Committee of New Zealand. General consultation with the Ngāi Tahu Research Committee of the University of Otago was carried out before the beginning of this research.

### PBMC isolation

Patients filled out a small survey detailing their current condition at the time of blood collection. Blood fractions were processed within on the same day. PBMCs were isolated from the whole blood by layering on Ficoll-Paque before separating plasma from PMBCs and other cells by centrifuging at 400×*g*. The PBMC layer was pelleted (100×*g*) through PBS and the resulting pellet resuspended in PBS and RNA later and stored at − 80 °C.

### DNA extraction

DNA was extracted from 200 μL of the PBMC fraction using the Illustra blood Genomic Prep Mini Spin Kit according to the manufacturer’s instructions. DNA was eluted in the kit’s EB buffer. Concentration was determined utilising the Qubit 2.0 fluorometer, following the Qubit dsDNA HS Assay Kit protocol [50].

### Reduced representation bisulphite sequencing

RRBS libraries were prepared as previously described [55–58]. Briefly, genomic DNA (500 µg) was digested with 160 units MSP1 restriction enzyme. Following end repair and adenylation of 3′ ends, adaptors were ligated to the DNA fragments. Bisulphite conversion was performed using the specifications of the EZ DNA methylation Kit. Semi-quantitative PCR was performed on the bisulphite converted DNA in order to determine the optimal amplification cycle needed for the final large scale PCR of the overall library. Following PCR amplification of the DNA, it was size-selected using a 6%(w/v) NuSieve Gel in order to extract the desired size (40–220 bp) for the RRBS libraries and to minimise adaptor contamination. Following purification and analysis of quality using a BioAnalyzer and Qubit measures, samples were further purified using AMPure XP Bead purification.

### DNA sequencing

The majority of the samples were sequenced through Custom Science; however, C012 and ME016 were sequenced through the Otago Genomics and Bioinformatics Facility. Following sequencing, the raw fastq files were checked for adaptor presence and trimmed. The data were aligned to the human genome version GRCh37/hg19 using Bismark bowtie alignment generating BAM files utilised in the differential methylation analysis.

### Statistical analyses

Analyses were performed in two parts, first using the DMAP analysis program run on a MAC OS X computer in order to investigate changes across fragments 40–220 bp in length and then in R Studio using the MethylKit package to investigate changes in methylation on a single cytosine basis.

DMAP used an ANOVA F test comparison between the patient and the control groups. A minimum of seven individuals per group had to have data for the fragment being compared to be included in the criteria. A raw *P* value threshold < 0.05 was established. The genomic features overlapping with the features were identified using the DMAP inbuilt Geneloc function and then with GenomicRanges with promoter overlaps (sourced from TxDb hg19 genomic data).

MethylKit performed a Fisher’s test on the pooled patient and control groups (with a minimum of seven individuals having methylation records at each cytosine in order for it to be included). A FDR-corrected *P* value threshold of < 0.05 was established. The genomic features overlapping with differential methylation were determined using TxDb hg19 genomic annotation data.

## Supplementary information

**Additional file 1:** Excel file containing additional information for this study including sheets; ‘Patient_Background’, ‘Symptom_Presentation’, ‘DMAP_Diff_Fragments’, ‘MethylKit_Diff_Cytosines’, ‘Cluster_Data’, ‘MethylKit_Promoter_Full’, ‘DMAP_Gene_Full’, ‘MethylKit_Gene_Full’, ‘DMAP_Pathways’, ‘MethylKit_Pathways’ and ‘Genelist_Overlap’.

**Additional file 2:** Figure S1. Box plots summarizing the age, weight and heights of the patients included in this study.

**Additional file 3:** Word document ‘Questionnaire’ containing the ME/CFS patient questionnaire that was completed by each patient at the onset of this study.

## Data Availability

The datasets generated and analysed during this current study are available in the GEO database NCBI (Accession Number GSE153667).

## Abbreviations

CpG: 5′-Cytosine-3′ phosphate-5′ Guanosine-3′
DMC: Differentially methylated cytosine
DMF: Differentially methylated fragment
DMAP: Differential methylation analysis program
ME/CFS: Myalgic Encephalomyelitis/Chronic Fatigue Syndrome
PBMC: Peripheral blood mononuclear cell
RRBS: Reduced representation bisulphite sequencing
