# Supplementary material for: Changes in DNA methylation profiles of myalgic encephalomyelitis/chronic fatigue syndrome patients reflect systemic dysfunctions
Source: Clin Epigenetics. 2020 Nov 4;12:167. doi: 10.1186/s13148-020-00960-z (PMC7641803; doi:10.1186/s13148-020-00960-z)
Supplement: Supplementary file 2 — Additional file 2: Figure S1. Box plots summarizing the age, weight and heights of the patients included in this study. [file 13148_2020_960_MOESM2_ESM.pdf]

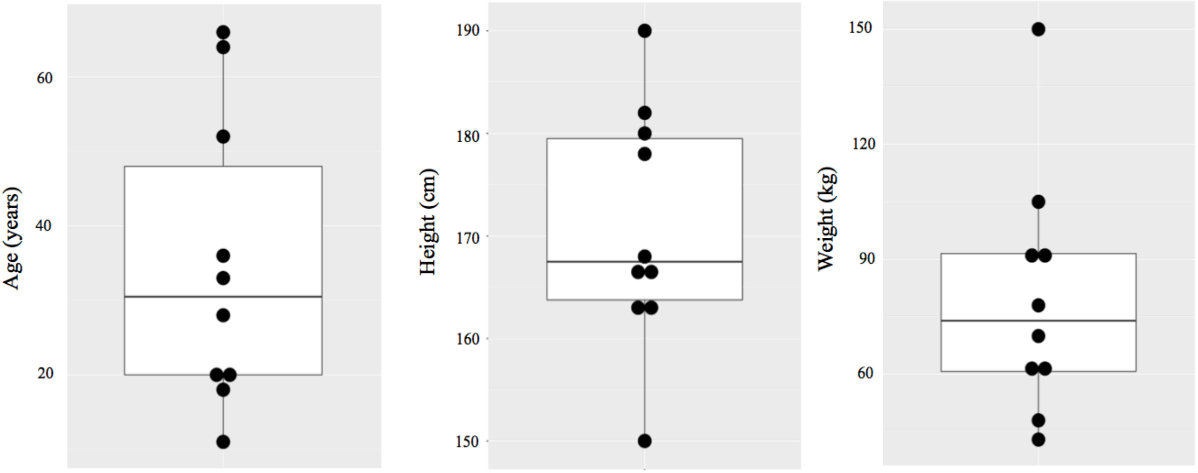

Supplementary Figure One. Boxplots showing the variation in age (years), weight (kg) and heights (cm) for the ME/CFS patient cohort.
