# Supplementary material for: Changes in DNA methylation profiles of myalgic encephalomyelitis/chronic fatigue syndrome patients reflect systemic dysfunctions
Source: Clin Epigenetics. 2020 Nov 4;12:167. doi: 10.1186/s13148-020-00960-z (PMC7641803; doi:10.1186/s13148-020-00960-z)
Supplement: Supplementary file 3 — Additional file 3: Word document ‘Questionnaire’ containing the ME/CFS patient questionnaire that was completed by each patient at the onset of this study. [file 13148_2020_960_MOESM3_ESM.docx]

Professor Warren Tate

Principal Investigator

Dept. of Biochemistry

University of Otago

PO Box 56, Dunedin 9054

[warren.tate@otago.ac.nz](mailto:warren.tate@otago.ac.nz)

***Developing a diagnostic blood test for ME/CFS – Confidential Questionnaire***

*Please provide the information requested – Tick boxes, where applicable*

Date: _______________ ID# (Internal Use):

|  |  | | |
| --- | --- | --- | --- |
| 1. Gender | □ Male  □ Female | | |
| 1. Age | ……………...Years | | |
| 1. Weight | ......................Kg or ……………. stones/ lb. | | |
| 1. Height | ......................m/cm or …………… feet/ in. | | |
| 1. Ethnic group (Tick more than one box, if necessary) | □ NZ European  □ Maori – region & iwi ……………………………………………  …………………………………………...  □ Pacific Island – specify ………………………………………..  □ Chinese  □ Indian  □ Other ……………………………………………………………. | | |
| 1. When do you think your ME/CFS symptoms began? | Approx. Month/Year: ………………………………. | | |
| 1. When were you diagnosed with ME/CFS? | Approx. Month/Year: ………………………………. | | |
| 1. Was there an initial triggering event for your ME/CFS? Please tick any appropriate boxes and provide additional detail, if possible, over the page. | □ An acute infection like glandular fever  □ Accident/ surgery  □ Severe physical stress  □ Severe emotional stress/ worry  □ Chemical exposure  □ Vaccination  □ Blood transfusion  □ A trip or vacation  □ Menopause  □ Not sure  Additional detail (optional): ………………………………………….  ………………………………………………………………………….  ………………………………………………………………………….  …………………………………………………………………………. | | |
| 1. Was your onset of ME/CFS sudden or gradual? | □ Sudden  □ Gradual | | |
| 1. If gradual how long did it take? | □ 1-4 weeks  □ 1-12 months  □ 1 or more years | | |
| 1. Your symptom progression | □ Became slowly worse  □ Stayed same  □ Fluctuated, without ever feeling better  □ Fluctuated, with periods of relapse (bad) and remission (no symptoms)  □ Became slowly better | | |
| 1. Factors contributing to your relapses (Tick all relevant boxes & provide brief details if possible) | □ Stress ……………………………………………………………  □ Physical exertion ……………………………………………….  □ Infections/ Allergies ……………………………………………  □ Vaccines ……...….……………………………………………..  □ Lack of Sleep …………………………………………………..  □ Alcohol …………………………………………………….........  □ Dietary …………………………………………………………..  □ Chemical ………………………………………………………..  □ Climate/ Seasonal changes …………………………….........  □ Menstrual Cycle ………………………………………………..  □ Other ……….……………………………………………………  …………………………………………………………………….  …………………………………………………………………….  ……………………………………………………………………. | | |
| 1. Number of relapses or set-backs in the last year & average duration | Number: ................. Duration: ................. | | |
| 1. The severity of your illness currently (Tick the appropriate boxes) | □ Mostly Bedridden  □ Mostly Housebound  □ Limited activity, not able to work  □ Work part-time (or equivalent)  □ Work full-time (or equivalent), but limited social life or other activities | | |
| 1. If you experienced a serious or persistent illness/ condition BEFORE the onset of your ME/CFS, please tick an appropriate box and specify the particular disorder(s), dates of onset and duration in the spaces provided. | □ Chronic infectious diseases ……………………………........... ………………………………………………………………………….  □ Psychological/ Psychiatric disorders ………………………….  …………………………………………………………………….  □ Heart/ lung disease ……………............................................. …………………………………………………………………………  □ Diabetes …………………………………………………………  …………………………………………………………………….  □ Hormonal disorders e.g. Menopause, Thyroid …………………………................................................................ ………………………………………………………………………..  □ Anaemia …………………………………………………………  ……………………………………………………………………  □ Cancer …………………………….......................................... ………………………………………………………………………..  □ Bowel disease ………………………………………………….  …………………………………………………………………….  □ Neurological disease ……………........................................... ………………………………………………………………………….  □ Sleep disorder ………………………………………………….  ……………………………………………………………………  □ Other e.g. Asthma ………………………………………………….................................  …………………………………………………………………………  ………………………………………………………………………… | | |
| 1. Indicate in the space provided if you have experienced any of the above illnesses/ conditions SINCE the onset of your ME/CFS, including name, date of onset and duration. | ……………………………………………………………………  ……………………………………………………………………  ……………………………………………………………………  ……………………………………………………………………  ……………………………………………………………………  ……………………………………………………………………  ……………………………………………………………………  …………………………………………………………………… | | |
|  |  | | |
| 1. Please list (with dates), in the spaces provided, any other significantly demanding/ stressful events that you have experienced both BEFORE and SINCE you got ME/CFS provided e.g. accidents, surgery, pregnancy. | Before ME: ……………………………………………………………………  ……………………………………………………………………  ……………………………………………………………………  ……………………………………………………………………  Since ME:  ……………………………………………………………………  …………………………………………………………………….  ……………………………………………………………………. | | |
| 1. If you have subjected yourself to heavy sustained alcohol use and/ or substance/ drug use, please tick an appropriate box and provide the date of onset and duration in the space provided. | | □ Alcohol …………...…………………………………………  ………………………………………………………………..  □ Substance/ drug …………………………………………... ………………………………………………………………..  □ Other ………………………………………………………..  ………………………………………………………………. | |
| 1. Outline briefly any medications/ therapies you are undertaking for ME/CFS | | | ………………………………………………………………  ………………………………………………………………  ………………………………………………………………  ………………………………………………………………  ………………………………………………………………  ……………………………………………………………….  ……………………………………………………………….  ………………………………………………………………  ………………………………………………………………. |

| 1. Outline briefly any medications/ therapies you are undertaking for non-ME/CFS related illnesses/ conditions/ allergies. | ………………………………………………………………  ………………………………………………………………  ………………………………………………………………  ………………………………………………………………  ………………………………………………………………  ……………………………………………………………….  ……………………………………………………………….  ………………………………………………………………  ………………………………………………………………. |
| --- | --- |

| 1. Is there a history of fibromyalgia/ ME/CFS in your family (including close relations)? If yes, please provide more details e.g. specify the illness/ family relation/ date of onset. | □ Yes  □ No  ………………………………………………………………  ………………………………………………………………  ………………………………………………………………  ……………………………………………………………….  ………………………………………………………………. |
| --- | --- |
|  |  |
| 1. Is there a history of auto-immune disease in your wider family e.g. lupus erythematosus, Sjögren’s syndrome, type 1- diabetes, rheumatoid arthritis or similar illness? If yes, please provide details in the space provided e.g. name of illness/ family relation/ date of onset. | □ Yes  □ No  ………………………………………………………………  ………………………………………………………………  ………………………………………………………………  ……………………………………………………………….  ……………………………………………………………….  ……………………………………………………………….  ………………………………………………………………. |
|  |  |

*The following chart relates to symptoms of your illness over the last year. Please fill in the chart, for each symptom, working from left to right.*

| **Symptom** | ***Frequency***  **How *often*** have you had this symptom?  For each symptom listed, **circle** a number from this 0-4 scale:  0 = none of the time  1 = some of the time  2 = about half of the time  3 = most of the time  4 = all of the time | ***Severity***  **How *much*** did this symptom bother you, when at its worst?  For each symptom listed, **circle** a number from this 0-4 scale:  0 = not at all  1 = mildly  2 = moderately  3 = severely  4 = very severely |
| --- | --- | --- |
| 1. Persistent fatigue | 0 1 2 3 4 | 0 1 2 3 4 |
| 1. Muscle weakness | 0 1 2 3 4 | 0 1 2 3 4 |
| 1. Exhausted easily from physical/ mental activity | 0 1 2 3 4 | 0 1 2 3 4 |
| 1. Recurrent flu-like malaise | 0 1 2 3 4 | 0 1 2 3 4 |
| 1. Sore throat/ swollen glands | 0 1 2 3 4 | 0 1 2 3 4 |
| 1. Physical effort makes you feel worse | 0 1 2 3 4 | 0 1 2 3 4 |
| 1. Mental effort makes you feel worse | 0 1 2 3 4 | 0 1 2 3 4 |
| 1. Intolerance to stressful events | 0 1 2 3 4 | 0 1 2 3 4 |
| 1. Recovery after too much activity takes much longer than before onset of ME/CFS | 0 1 2 3 4 | 0 1 2 3 4 |
| 1. Difficulty in thinking | 0 1 2 3 4 | 0 1 2 3 4 |
| 1. Short-term memory loss | 0 1 2 3 4 | 0 1 2 3 4 |
| 1. Persistent headaches | 0 1 2 3 4 | 0 1 2 3 4 |
| 1. Aching eyes | 0 1 2 3 4 | 0 1 2 3 4 |
| 1. Blurred vision | 0 1 2 3 4 | 0 1 2 3 4 |
| 1. Muscle pain | 0 1 2 3 4 | 0 1 2 3 4 |
| 1. Spinal pain | 0 1 2 3 4 | 0 1 2 3 4 |
| 1. Joint pain | 0 1 2 3 4 | 0 1 2 3 4 |
| 1. Abdominal pain | 0 1 2 3 4 | 0 1 2 3 4 |
| 1. Nausea and/or bloating and/or bowel upsets | 0 1 2 3 4 | 0 1 2 3 4 |
| 1. Abnormal sleep patterns (insomnia/ prolonged sleep) | 0 1 2 3 4 | 0 1 2 3 4 |
| 1. Un-refreshed sleep | 0 1 2 3 4 | 0 1 2 3 4 |
| 1. Increased sensitivity to light/ sound/ odours. | 0 1 2 3 4 | 0 1 2 3 4 |

*The following chart relates to symptoms of your illness over the last year. Please fill in the chart, for each symptom, working from left to right.*

| **Symptom** | ***Frequency***  **How *often*** do you have this symptom?  For each symptom listed, **circle** a number from this 0-4 scale:  0 = none of the time  1 = some of the time  2 = about half of the time  3 = most of the time  4 = all of the time | | ***Severity***  **How *much*** does this symptom bother you, when at its worst?  For each symptom listed, **circle** a number from this 0-4 scale:  0 = none of the time  1 = mildly  2 = moderately  3 = severely  4 = very severely |
| --- | --- | --- | --- |
| 1. More frequent viral-type infections with longer recovery times | 0 1 2 3 4 | | 0 1 2 3 4 |
| 1. Urinary problems e.g. pain/ frequency. | 0 1 2 3 4 | 0 1 2 3 4 | |
| 1. Increased intolerance of foods, medications, chemicals/ alcohol | 0 1 2 3 4 | 0 1 2 3 4 | |
| 1. Light-headed/ dizzy, unsteady on your feet | 0 1 2 3 4 | 0 1 2 3 4 | |
| 1. Heart irregularities | 0 1 2 3 4 | 0 1 2 3 4 | |
| 1. Breathing problems e.g. laboured breathing | 0 1 2 3 4 | 0 1 2 3 4 | |
| 1. Intolerance of temperature extremes | 0 1 2 3 4 | 0 1 2 3 4 | |
| 1. Weight fluctuations | 0 1 2 3 4 | 0 1 2 3 4 | |

| 1. Please add any other symptoms you have experienced and feel should be included. If possible assign a “frequency” and a “severity” number as above. | ……………………………………………………………….........  …………………………………………………………………….  …………………………………………………………………….  …………………………………………………………………….  …………………………………………………………………….  …………………………………………………………………….  …………………………………………………………………….  …………………………………………………………………….  …………………………………………………………………….  ……………………………………………………………………. |
| --- | --- |
